# Spring Door Closer Entrapment of the Upper Eyelid: A Pediatric Periocular Foreign‐Body Injury With a Favorable Functional Outcome—A Case Report

**DOI:** 10.1002/ccr3.73348

**Published:** 2026-08-17

**Authors:** Mac‐Cauley Harrison, Gladys Fordjuor, Benjamin Abaidoo, Naa Naamuah Tagoe, Hafisatu Gbadamosi, Lauretta Pinamang Owusu‐Asare, Haruna Muniratu, Joseph Amegashitsi, Kofi Obeng Amoafo, Emmanuel Kitcher, Dorothy Hinson, Anna Kissiwaa Badu, Abigail Ofeibea Addy, Egote Constance Amuzuah

**Affiliations:** ^1^ Eye Department Korle Bu Teaching Hospital Accra Ghana; ^2^ Ophthalmology Unit, Department of Surgery University of Ghana Medical School Accra Ghana; ^3^ Department of Radiology Korle Bu Teaching Hospital Accra Ghana; ^4^ Department of Anaesthesia Korle Bu Teaching Hospital Accra Ghana

**Keywords:** case report, eyelid foreign body, pediatric ocular trauma, spring door closer, trapdoor spring

## Abstract

Ocular trauma in children is an important cause of avoidable visual morbidity, with potential psychosocial, educational, and quality‐of‐life consequences. Domestic spring door closers, commonly called “trapdoor springs” in Ghana, may cause significant periocular injury when an exposed hook is accidentally encountered; however, this mechanism is rarely described in the ophthalmic literature. We report a 10‐year‐old girl who presented shortly after a household accident with a metallic spring door closer hook embedded in the left upper eyelid. Marked eyelid edema, mechanical ptosis, pain, tearing, and distress prevented reliable initial assessment of visual acuity, pupillary responses, ocular motility, and the anterior and posterior segments of the affected eye. Non‐contrast orbital computed tomography (CT) demonstrated a metallic foreign body confined to the upper eyelid and periocular soft tissues, without globe penetration, orbital wall fracture, or retrobulbar hemorrhage. The foreign body was removed in theater under general anesthesia by controlled disengagement without forceful traction. Intraoperative findings included corneal and conjunctival abrasions and a 6 mm conjunctival laceration, which was repaired with 8‐0 Vicryl absorbable suture. Postoperative care included tetanus prophylaxis, analgesia, topical antibiotic‐corticosteroid therapy, lubrication, and scheduled review. At 2 months, unaided visual acuity was 6/6 and intraocular pressure was 12 mmHg in both eyes; ocular motility was full, the anterior and posterior segments were normal, and there was no evidence of globe injury. This case highlights the importance of prompt CT localization, avoidance of unsafe bedside traction, controlled theater removal with ocular‐surface protection, meticulous repair, structured follow‐up, and household safety measures.

## Key Clinical Message

Spring door closer hooks can entrap the eyelid and conceal globe injury. When examination is limited by pain, edema, or mechanical ptosis, prompt CT localization, avoidance of forceful bedside traction, controlled theater removal, and structured follow‐up can support a favorable functional and cosmetic outcome.

## 1 Introduction

Ocular trauma remains an important and preventable cause of visual morbidity worldwide. Children are particularly vulnerable because injuries frequently occur during play, domestic activities, and unsupervised interaction with household objects. Although many pediatric ocular injuries are not life‐threatening, they may threaten vision and have important psychosocial, educational, quality‐of‐life, and economic consequences for affected children and their families [1, 2, 3, 4, 5]. Studies from Ghana have also identified ocular trauma as an important public health problem in both pediatric and general populations [6, 7, 8, 9].

Periocular foreign‐body injuries may appear deceptively localized while obscuring potentially serious globe or orbital involvement, particularly when pain, eyelid edema, blepharospasm, or mechanical ptosis prevents reliable bedside examination. Hooked foreign bodies require particular caution because blind or forceful traction may enlarge the wound or cause additional injury to the eyelid, conjunctiva, ocular surface, or globe. Reports of eyelid‐hook and fish‐hook injuries emphasize the importance of careful assessment, controlled removal, and prevention of avoidable domestic and recreational injuries [10, 11, 12].

**FIGURE 1:**
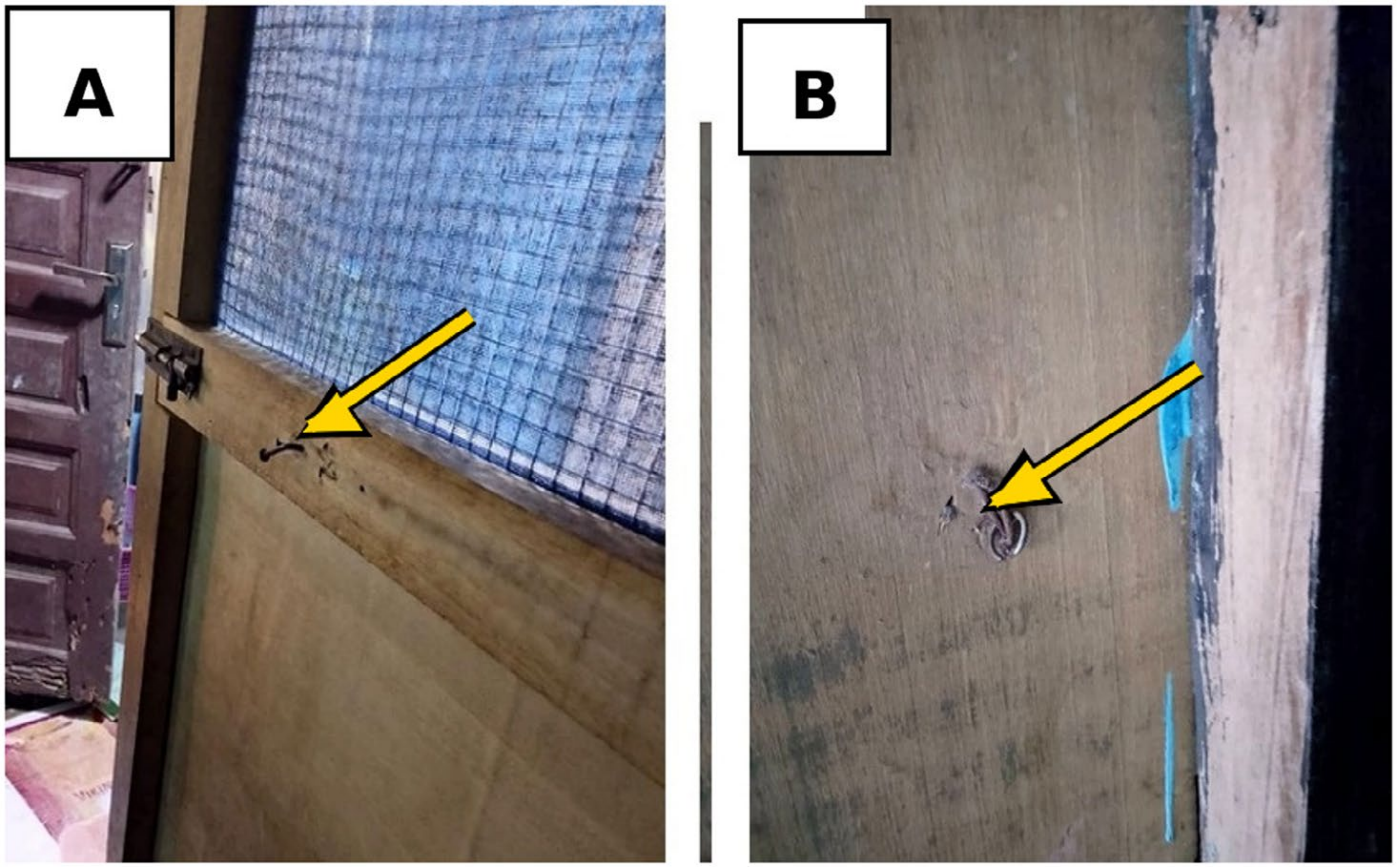
Spring door closer mechanism at the patient's injury setting. (A) Wider view of the doorway showing the exposed terminal metal hook identified by the yellow arrow. (B) Close‐up of the same protruding hook identified by the yellow arrow. The arrows identify the component that caught and became embedded in the patient's left upper eyelid while it remained connected to the tension‐spring mechanism. This continuing attachment meant that pulling on the hook could have enlarged the eyelid wound or caused additional ocular‐surface injury.

**FIGURE 2:**
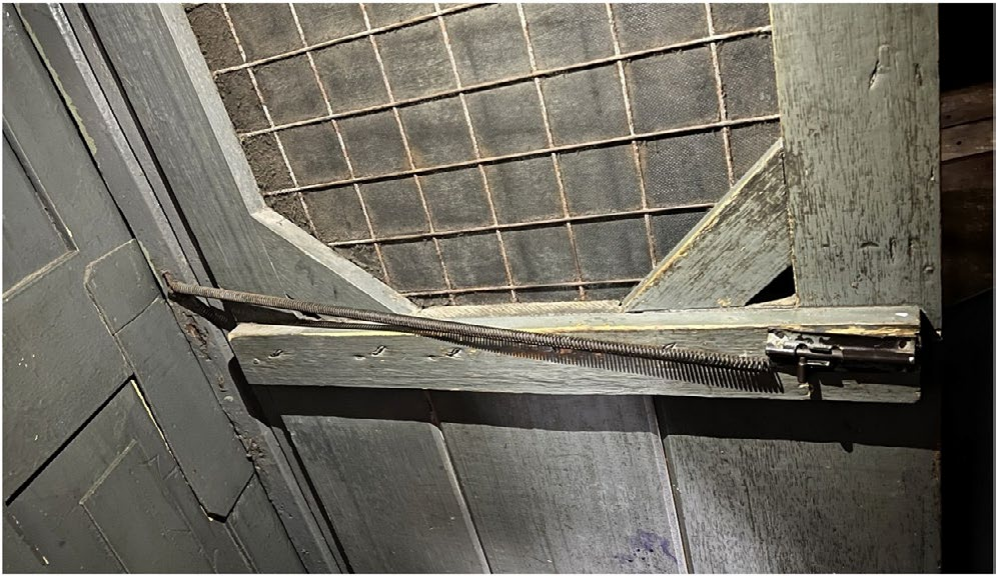
Representative household installation of a spring door closer similar to the mechanism involved in the injury, showing the complete tension‐spring assembly mounted between the door and frame. The photograph was taken by the corresponding author and does not depict the patient's injury setting.

Spring door closers, locally referred to as “trapdoor springs” in Ghana, use a spring‐loaded mechanism to close doors. An exposed protruding hook may accidentally catch periocular soft tissue. The spring door closer mechanism from the patient's injury setting is shown in Figure 1. A representative household installation of a similar spring door closer, demonstrating the complete tension‐spring assembly, is shown in Figure 2. We conducted a focused literature search of PubMed and Google Scholar using the terms “spring door closer,” “door closer,” “door spring,” and “trapdoor spring,” combined with “eyelid,” “eye,” “ocular,” and “periocular.” We did not identify a previously published report specifically describing eyelid entrapment caused by a spring door closer or trapdoor spring mechanism, although related domestic and recreational eyelid‐hook injuries have been reported [10, 11]. When clinical examination of the globe is unsafe or incomplete in the presence of a metallic periocular foreign body, computed tomography (CT) can define the object's location and its relationship to the globe and orbital structures [13, 14, 15]. Magnetic resonance imaging should be avoided until metallic foreign material has been excluded [13].

We report an unusual pediatric domestic periocular injury in which a spring door closer hook became embedded in the upper eyelid. Despite a dramatic presentation and initially limited ocular examination, early CT localization, controlled theater removal, careful ocular‐surface inspection and repair, and structured follow‐up resulted in a favorable functional and cosmetic outcome. The case provides practical lessons in assessment, imaging, surgical planning, postoperative care, and prevention of similar household injuries.

## 2 Case Presentation

### 2.1 Patient Information and History

A 10‐year‐old girl with no known previous ocular history presented to the emergency eye unit shortly after a household accident. According to her caregivers, a spring door closer, locally referred to as a “trapdoor spring,” caught her left upper eyelid, leaving the coiled spring and hook embedded in the lid. She experienced immediate pain, tearing, and inability to open the affected eye. No attempt was made at home to remove the object forcefully. The spring door closer mechanism from the patient's injury setting is shown in Figure 1. Figure 2 shows a representative household installation of a similar spring door closer to demonstrate the complete spring assembly.

### 2.2 Clinical Findings at Presentation

**FIGURE 3:**
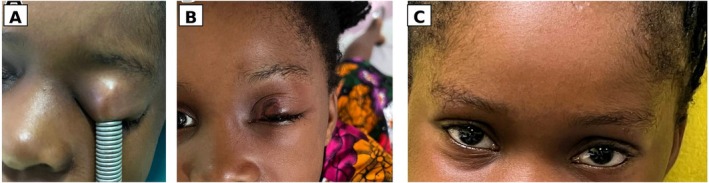
Clinical course of the left upper‐eyelid injury: (A) initial presentation showing the spring door closer hook impaled in the upper eyelid, with marked edema and mechanical ptosis; (B) postoperative Day 1 showing reduced swelling and a clean healing eyelid wound; (C) 2‐month review showing a healed eyelid with good contour and function. Images have been cropped to the periocular region to minimize patient identifiability.

On Day 0, examination showed a metallic spring door closer hook impaled in the left upper eyelid, with marked eyelid edema, mechanical ptosis, clotted blood at the presumed entry site, pain, tearing, and inability to open the affected eye (Figure 3A). No obvious eyelid‐margin laceration was identified on initial inspection; however, assessment was limited by swelling, clotted blood, and the embedded spring.

Visual acuity in the left eye could not be assessed safely and reliably because the upper eyelid was mechanically entrapped and ptotic. The child was in pain and distressed, and forced eyelid opening was avoided to prevent additional eyelid trauma or possible injury to an unassessed globe. Reliable assessment of pupillary reactions, relative afferent pupillary defect, extraocular movements, and the anterior and posterior segments of the affected eye could therefore not be completed at the bedside. No abnormality was identified in the right eye on the available examination, and a focused neurological assessment revealed no abnormality.

Intravenous access was established, and Ringer's lactate and intravenous paracetamol were administered. The foreign body was left undisturbed pending imaging and controlled removal because its relationship to the globe and orbit had not yet been established.

### 2.3 Diagnostic Assessment

**FIGURE 4:**
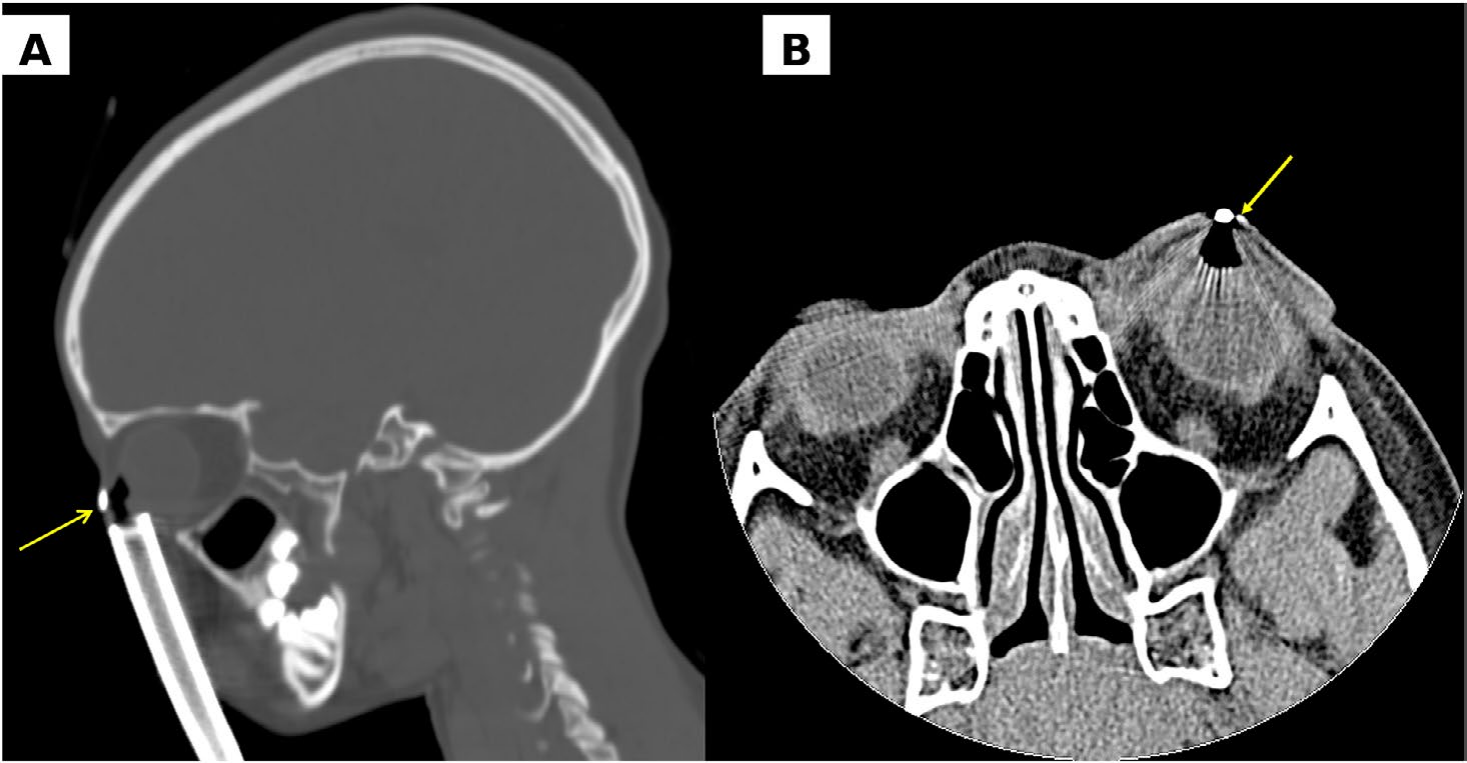
Non‐contrast orbital computed tomography images: (A) sagittal reformatted bone‐window image; (B) axial soft‐tissue‐window image. Yellow arrows indicate the metallic foreign body embedded within the left upper eyelid and confined to the periocular soft tissues. The globe is intact, with no orbital wall fracture or retrobulbar hemorrhage.

**FIGURE 5:**
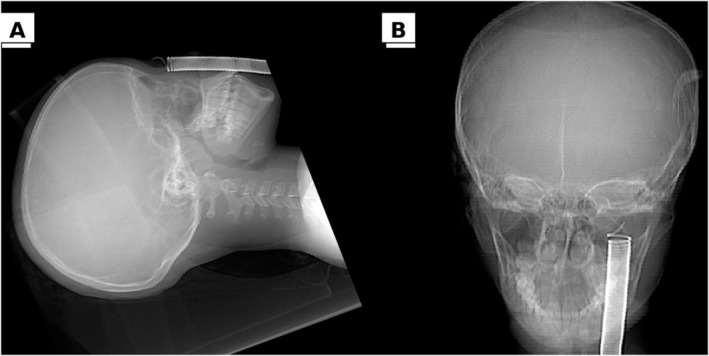
Computed tomography scout images: (A) lateral view; (B) anteroposterior view, demonstrating the coiled spring and hooked tip located anterior to the left orbit.

Because the globe could not be fully and safely assessed clinically and the foreign body was metallic, non‐contrast orbital computed tomography was performed to determine its location and relationship to the globe and orbital structures. The scan demonstrated a metallic foreign body traversing the soft tissues of the left upper eyelid, with associated eyelid thickening and periocular edema (Figure 4A,B). The foreign body was confined to the upper eyelid and periocular soft tissues. Both globes were intact, with no orbital wall fracture or retrobulbar hemorrhage. A small focus of air was present anteriorly between the eyelid and ocular surface, consistent with local periocular soft‐tissue emphysema. Computed tomography scout images demonstrated the coiled spring and hooked tip anterior to the left orbit (Figure 5A,B). A timeline of the presentation, intervention, and clinical outcome is provided in Table 1.

**TABLE 1:** Timeline of presentation, intervention and clinical outcome.

Date/interval	Events and findings
Day 0 (presentation)	Spring door closer hook impaled in the left upper eyelid, with marked edema, mechanical ptosis, clotted blood at the presumed entry site, pain, tearing and inability to open the affected eye. Visual acuity, pupillary reactions, relative afferent pupillary defect, extraocular movements, and anterior‐ and posterior‐segment findings could not be reliably assessed. Intravenous Ringer's lactate and paracetamol were administered. The foreign body was left undisturbed pending imaging and controlled removal
Day 0 (imaging)	Non‐contrast orbital CT showed a metallic foreign body confined to the left upper eyelid and periocular soft tissues. Both globes were intact, with no orbital wall fracture or retrobulbar hemorrhage
Day 0 (theater)	Exploration and foreign‐body removal under general anesthesia; protective lid guard placement; controlled disengagement without forceful traction; identification of corneal abrasions, conjunctival abrasions and a 6 mm conjunctival laceration; irrigation and hemostasis; conjunctival repair with 8‐0 Vicryl absorbable suture; eye padding and tetanus prophylaxis
Postoperative Day 1	Reduced periocular edema and clean eyelid wound. Visual acuity and intraocular pressure were not formally recorded because residual swelling and discomfort precluded reliable testing. Topical treatment, lubrication, analgesia and wound care were continued
Postoperative Week 1	Mild residual eyelid edema, clear cornea and mild conjunctival injection. Unaided visual acuity was 6/9 in the left eye, improving to 6/6 with pinhole, and 6/6 in the right eye. Intraocular pressure was 16 mmHg in the left eye and 12 mmHg in the right eye
Postoperative Month 1	Healed eyelid wound with mild residual ptosis and no evidence of infection. Unaided visual acuity was 6/6 in both eyes. Intraocular pressure was 15 mmHg in the left eye and 13 mmHg in the right eye. Maxitrol ointment was discontinued
Two‐month review	Healed eyelid with good contour and function. Unaided visual acuity was 6/6 and intraocular pressure was 12 mmHg in both eyes. Ocular motility was full, and there was no evidence of globe injury. Refresh Tears was discontinued

### 2.4 Therapeutic Intervention

Bedside extraction was avoided because the globe could not be assessed safely and traction on the hooked metallic spring could have caused additional eyelid or ocular injury. After informed consent, exploration of the left eye and removal of the foreign body were performed under general anesthesia. The patient was positioned supine, and the periocular region was cleaned and draped using sterile technique.

**FIGURE 6:**
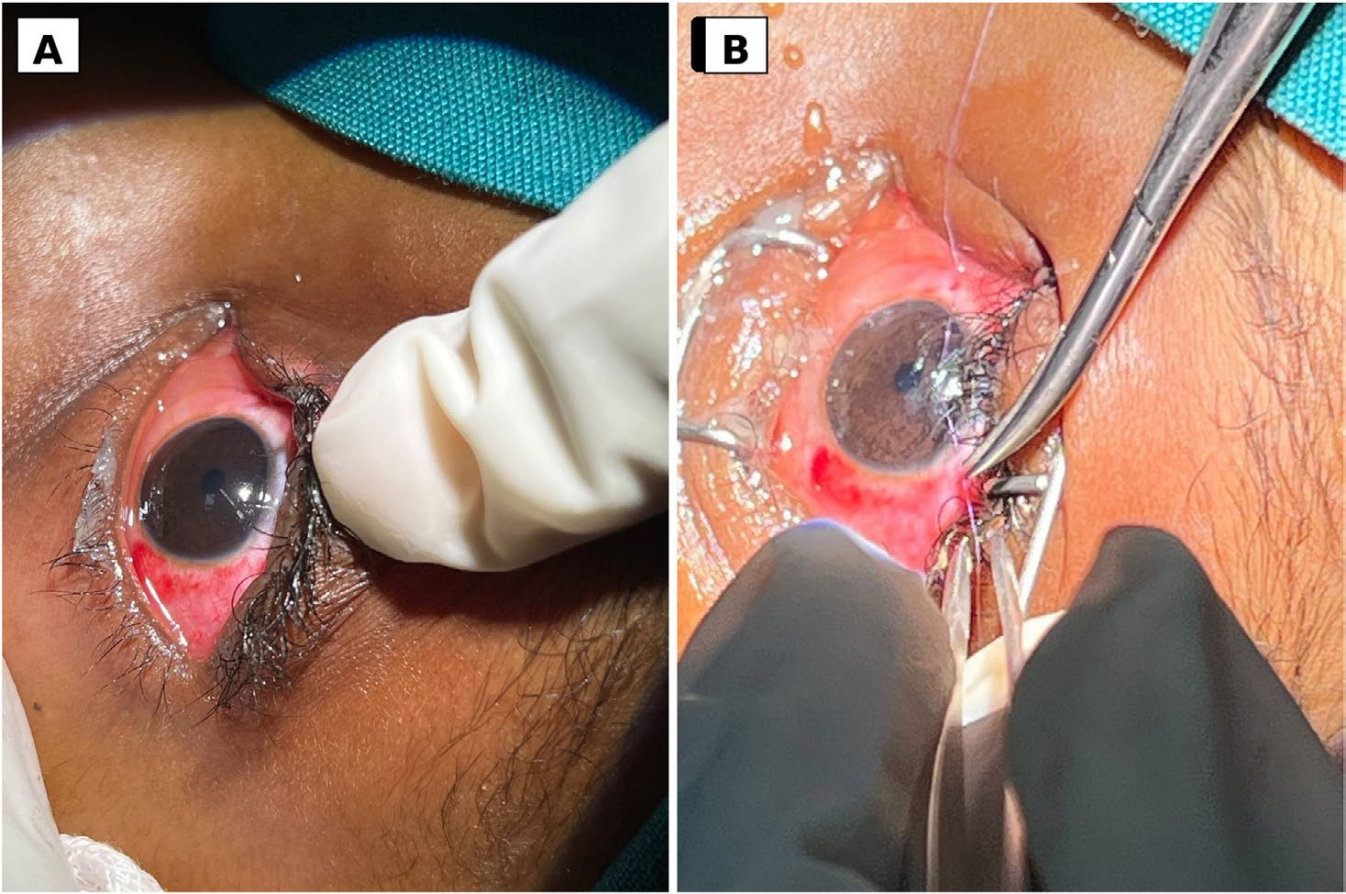
Intraoperative photographs: (A) ocular‐surface inspection following controlled removal of the foreign body, showing conjunctival hyperemia and an intact globe; (B) repair of the associated 6 mm conjunctival laceration with 8‐0 Vicryl absorbable sutures.

In theater the direction of entry of the hook and its relationship to the upper eyelid and palpebral conjunctiva were assessed. The upper eyelid was supported, and a lid guard was placed over the ocular surface to protect the cornea and globe during manipulation. The spring hook was gently disengaged from the palpebral conjunctival and upper‐eyelid tissues under direct visualization, avoiding forceful traction and additional tissue injury. Following removal, the upper eyelid was everted and the ocular surface was carefully inspected (Figure 6A).

Intraoperative findings included corneal abrasions, conjunctival abrasions and an associated 6 mm conjunctival laceration. The globe was intact, with no evidence of corneal or scleral perforation. The wound and ocular surface were thoroughly irrigated, hemostasis was achieved, and the conjunctival laceration was repaired with 8‐0 Vicryl absorbable suture (Figure 6B). The eye was cleaned and padded at the end of the procedure. No intraoperative complication was documented.

Postoperatively, tetanus prophylaxis was administered, and the patient was admitted for observation. Intravenous fluids and intravenous paracetamol were continued for 24 h. Analgesia was subsequently changed to weight‐adjusted oral paracetamol and ibuprofen three times daily. Topical treatment was prescribed for the affected left eye only: Maxitrol eye drops (neomycin, polymyxin B and dexamethasone) four times daily for 1 week, followed by gradual tapering over 1 month and discontinuation; Maxitrol eye ointment at night for 1 month; and Refresh Tears lubricant eye drops four times daily for 2 months. No systemic oral antibiotic was administered.

### 2.5 Follow‐Up and Outcomes

On postoperative Day 1, the periocular swelling had begun to subside and the eyelid wound was clean (Figure 3B). Visual acuity and intraocular pressure were not formally recorded because residual eyelid swelling and discomfort precluded reliable testing. Topical antibiotic‐corticosteroid therapy, lubrication, analgesia, and wound care were continued.

At postoperative Week 1, mild residual eyelid edema, a clear cornea and mild conjunctival injection were present. Unaided visual acuity was 6/9 in the left eye, improving to 6/6 with pinhole, and 6/6 in the right eye. Intraocular pressure was 16 mmHg in the left eye and 12 mmHg in the right eye. Maxitrol drops were tapered as planned, while lubrication and wound care were continued.

At postoperative Month 1, the eyelid wound had healed, with mild residual ptosis and no evidence of infection. Unaided visual acuity was 6/6 in both eyes. Intraocular pressure was 15 mmHg in the left eye and 13 mmHg in the right eye. Maxitrol ointment was discontinued at 1 month.

At the 2‐month review, the eyelid had healed with good contour and function, no clinically apparent lid malposition and an adequately protected ocular surface (Figure 3C). Unaided visual acuity was 6/6 and intraocular pressure was 12 mmHg in both eyes. The pupils were round, central and reactive, with no relative afferent pupillary defect. Ocular motility was full. The cornea was clear, the anterior segment was quiet, and posterior‐segment examination showed an attached retina, a vertical cup‐disc ratio of 0.3, and normal macula and retinal vessels. There was no evidence of globe injury. Refresh Tears was discontinued at 2 months.

## 3 Discussion

Pediatric ocular trauma may arise from overlooked mechanical hazards in the home [1, 2, 3, 4, 5, 6, 7, 8, 9]. This case illustrates a distinctive but preventable mechanism: an exposed hook connected to a tension spring entrapped the upper eyelid, while pain, edema and mechanical ptosis precluded reliable examination of the globe. The clinical significance lies not only in the depth of tissue penetration but also in the object's hooked configuration and continued attachment to the spring, which could have enlarged the wound or redirected the hook towards the conjunctiva, ocular surface or globe if traction had been applied. Although eyelid‐hook and fish‐hook injuries have been described, our focused literature search did not identify a previous report of upper‐eyelid entrapment specifically caused by a spring door closer [10, 11].

Previously reported hooked and penetrating periocular foreign‐body injuries demonstrate a spectrum of severity. Omar and Salleh described a rubber‐string metal hook that pierced the upper eyelid from the conjunctival surface without globe involvement; reverse tracking enabled removal with satisfactory healing [10]. Levy and Lifshitz reported an upper‐eyelid fish‐hook injury removed using a back‐out technique without complication [11]. Like the present case, these two injuries were confined to the eyelid and had favorable outcomes after controlled removal. In contrast, Choovuthayakorn et al. described a fish hook that traversed the eyelid and globe, lodged in the ciliary body and caused a localized retinal detachment requiring staged surgical management [16]. Ben Abdessalem et al. reported an intraorbital ballpoint‐pen foreign body in a 2‐year‐old child whose severe eyelid edema prevented reliable examination; CT localized the object and guided removal through an anterior orbitotomy [17]. Zulhisham and Shatriah reported a retained periorbital foreign body diagnosed 2 months after injury, illustrating the risk of delayed recognition [18].

Together, these reports show that the external appearance of a periocular wound may not reliably indicate the depth or severity of injury. Management should be guided by the object's configuration and trajectory, the completeness of the ocular examination, the child's ability to cooperate, and the possibility of occult globe or orbital involvement. Blind or forceful extraction should therefore be avoided, and cross‐sectional imaging should be considered when examination is incomplete or deeper extension cannot be excluded.

When the globe cannot be examined safely, imaging can help convert diagnostic uncertainty into a safer operative plan. Non‐contrast CT is the preferred initial modality for suspected metallic periocular or orbital foreign bodies because it can rapidly define location, depth and relationships to the globe and orbital structures while identifying associated fracture, emphysema or hemorrhage [13, 14, 15, 19]. In this patient, CT localization supported controlled theater management, but it did not remove the need for direct ocular‐surface inspection because clinically important abrasions or lacerations could remain concealed by the entrapped eyelid. Magnetic resonance imaging should be avoided until metallic foreign material has been excluded [13].

The operative setting and technique should minimize secondary injury. General anesthesia was appropriate in this distressed child because it provided analgesia, immobility and controlled exposure while allowing immediate management of any occult ocular injury. The essential principles were support of the eyelid, protection of the ocular surface with a lid guard, direct visualization and disengagement without forceful traction. These principles are consistent with the controlled reverse‐tracking or back‐out approaches used in superficial eyelid‐hook injuries [10, 11], whereas globe‐penetrating or deeper orbital injuries may require staged intraocular surgery or orbitotomy [16, 17]. Careful eversion and inspection after removal are essential because an intact globe may coexist with corneal, conjunctival or eyelid injury requiring treatment.

Postoperative care should address infection prevention, ocular‐surface protection, pain and healing, while follow‐up assesses wound integrity, residual ptosis, lid malposition, exposure keratopathy, visual recovery and intraocular pressure during topical corticosteroid use [12, 14, 20]. The progressive restoration of 6/6 unaided visual acuity, normal intraocular pressure, full ocular motility and good eyelid function supports a favorable short‐term outcome after timely localization, controlled removal, ocular‐surface protection and repair.

The case also has a practical prevention message. Exposed or poorly positioned hooks on spring door closers may be overlooked hazards in homes, schools and childcare environments. Caregivers should discourage children from playing with door‐closing springs, and damaged or protruding hooks should be repaired, shielded or replaced. Families should also be advised not to pull a hooked periocular object before professional assessment.

This report is limited by its single‐case design and 2‐month follow‐up; these limitations restrict generalizability and preclude estimation of the frequency or full range of outcomes associated with this mechanism. The initial visual acuity and complete ocular examination could not be documented because safe assessment was prevented by mechanical eyelid entrapment. Nevertheless, the case provides practical lessons regarding diagnostic caution, imaging, controlled extraction, post‐removal inspection, structured follow‐up and prevention in pediatric periocular trauma.

## 4 Conclusion

Spring door closer entrapment of the upper eyelid is an unusual but clinically important form of pediatric domestic trauma. When eyelid entrapment prevents reliable examination of the globe, prompt CT localization, avoidance of forceful bedside traction, controlled theater removal with ocular‐surface protection, careful inspection and repair, and structured follow‐up can support a favorable functional and cosmetic outcome. Repairing or shielding exposed spring hooks and improving caregiver awareness may help prevent similar injuries.

## 5 Patient Perspective

At the 2‐month review, the child and her caregivers expressed satisfaction with the functional and cosmetic outcome.

## Author Contributions

**Mac‐Cauley Harrison:** writing – review and editing, writing – original draft, project administration, conceptualization. **Gladys Fordjuor:** conceptualization, supervision. **Haruna Muniratu:** resources. **Naa Naamuah Tagoe:** conceptualization, supervision. **Benjamin Abaidoo:** supervision, project administration. **Joseph Amegashitsi:** resources. **Dorothy Hinson:** data curation. **Hafisatu Gbadamosi:** writing – original draft, supervision. **Lauretta Pinamang Owusu‐Asare:** writing – original draft, writing – review and editing. **Kofi Obeng Amoafo:** resources. **Emmanuel Kitcher:** data curation. **Anna Kissiwaa Badu:** data curation. **Egote Constance Amuzuah:** resources. **Abigail Ofeibea Addy:** data curation.

## Funding

The authors have nothing to report.

## Ethics Statement

Institutional ethics approval was not required for this single case report in accordance with institutional policy.

## Consent

Written informed consent for publication of the clinical details and accompanying images was obtained from the patient's parent or legal guardian. Assent for publication was also obtained from the child.

## Conflicts of Interest

The authors declare no conflicts of interest.

## Data Availability

All data relevant to this case are included in the article. Additional anonymized information may be available from the corresponding author on reasonable request, subject to institutional and ethical restrictions.
